# Top 50 Highly Cited Publications in Cleft Lip: A 100-Year Bibliometric Analysis Review

**DOI:** 10.7759/cureus.97884

**Published:** 2025-11-26

**Authors:** Abdulmohsen E Al Mulhem, Hanouf A Alangari, Lama N Alanazi, Abdullah Omar H Ahmed, Lama A Alkhwildi, Yara K Alwathnani, Abbas A Alhammad, Ahmed S Albagawi, Renad S Bakhamis, Nora M Alzoum, Ghada H Postaji, Rahaf M AlTurkistani, Heba Y Alojail

**Affiliations:** 1 College of Medicine, King Faisal University, Al-Ahsa, SAU; 2 College of Medicine, Imam Mohammad Ibn Saud Islamic University (IMSIU), Riyadh, SAU; 3 Collage of Medicine, King Faisal University, Al-Ahsa, SAU; 4 College of Medicine, Taibah University, Al-Madinah Al-Munawwarah, SAU; 5 College of Medicine, Arabian Gulf University, Manama, BHR; 6 College of Medicine, Umm Al-Qura University, Makkah Al-Mukarramah, SAU; 7 College of Medicine, Princess Nourah bint Abdulrahman University, Riyadh, SAU; 8 College of Medicine, Ibn Sina National College, Jeddah, SAU; 9 Department of Dermatology, College of Medicine, King Faisal University, Al-Ahsa, SAU

**Keywords:** bibliometric analysis, cheiloplasty, cleft lip repair, review article, surgical techniques

## Abstract

One of the most prevalent facial abnormalities in children is a cleft lip, affecting approximately one in 700 live births worldwide, which poses a serious problem for craniofacial surgeons. Over the past decades, cleft lip repair (cheiloplasty) has witnessed significant advances in surgical techniques, functional restoration, and aesthetic outcomes. This study conducted a bibliometric analysis of the 50 most cited articles in cleft lip surgery published over the previous 100 years, aiming to identify influential research, key trends, and leading contributors in the field. A structured search was carried out using the Web of Science database with specific keywords. Articles were selected based on citation count, excluding non-peer-reviewed or irrelevant publications. Extracted variables included annual citation frequency, study type, level of evidence, and use of patient-reported outcome measures (PROMs). Moreover, the analysis revealed that the majority of high-impact research was published during the 1990s and 2000s. Review articles and retrospective studies dominated the literature, while randomized clinical trials (RCTs) were relatively rare. *Plastic and Reconstructive Surgery* emerged as the most cited journal. Frequently addressed topics included surgical techniques, nasal deformity correction, and bilateral cleft lip repair. Finally, this study offers a comprehensive overview of the evolution of cleft lip research and underscores the need for more prospective, high-quality studies to advance clinical outcomes and improve surgical care.

## Introduction and background

**Figure 1:**
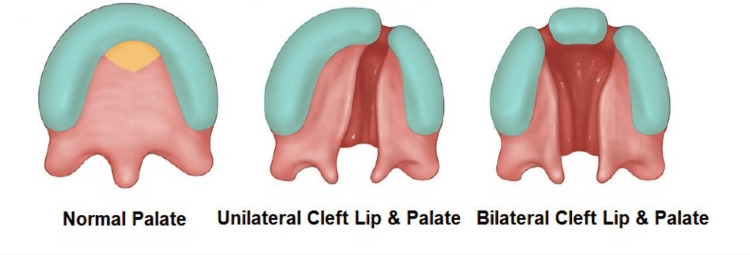
Normal embryologic palatal fusion compared with unilateral and bilateral cleft lip and palate development Source: [4]

A cleft lip forms when the upper lip does not develop completely during early fetal growth, leaving an opening or gap [1]. The severity of a cleft lip can vary widely, from a minor notch in the lip to a more pronounced gap that might extend into the nose. It can occur on one side of the lip (unilateral) or both sides (bilateral) (Figure 1) [2]. It is a prevalent facial malformation in pediatrics and presents significant challenges for craniofacial surgeons [3]. 

Globally, cleft lip with or without cleft palate affects approximately one in 700 live births, although prevalence varies by region and ethnicity. In the United States, approximately one in every 2,800 babies is born with this condition [5,6]. For children born with cleft lips, the impact often goes beyond appearance. They face challenges in feeding, speech, hearing, and psychosocial development, creating a substantial burden for both the child and their family. Addressing these challenges typically requires multidisciplinary care that extends beyond surgery, including specialized feeding support, speech and language therapy, audiologic monitoring, dental and orthodontic management, and psychosocial counseling, all of which contribute to long-term functional and developmental outcomes [6]. 

**Figure 2:**
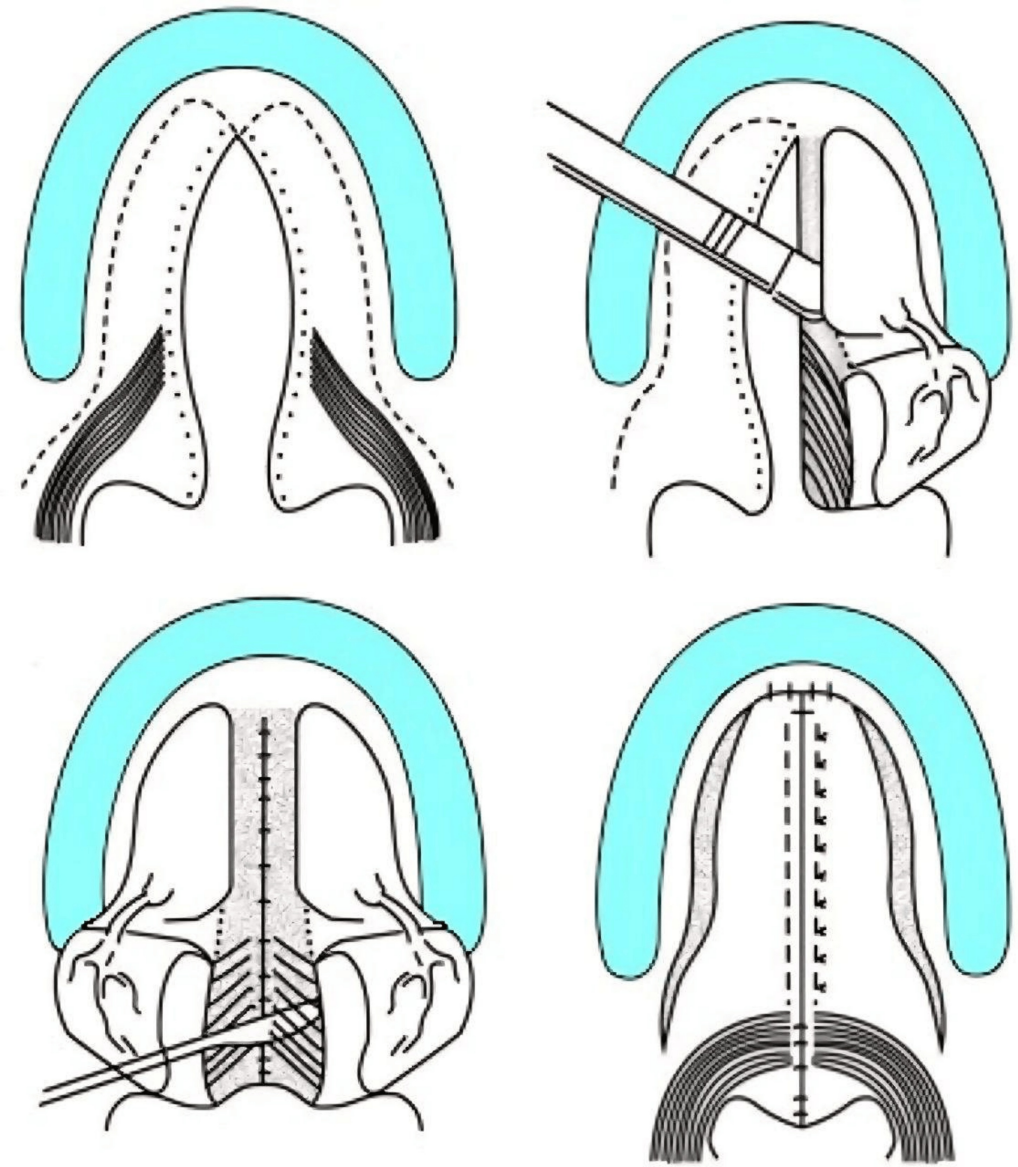
Surgical steps in bilateral mucoperiosteal flap elevation and midline closure for primary cleft palate repair Source: [7]

Surgical repair, known as cleft lip repair or cheiloplasty, is a critical step in the restoration of form and function in this condition. The procedure involves making precise incisions on either side of the cleft to create tissue flaps from the skin, muscle, and mucosa. These flaps are carefully repositioned and sutured together to close the gap, restoring the natural shape and function of the lip and nose (Figure 2) [1]. 

The primary goals of this surgery are to improve lip appearance, help the child develop normal speech, and improve overall quality of life [8]. Cleft lip surgery has a long history of progress and innovation, with each generation of surgeons building on the work of those who came before them. Over time, techniques have become more precise and tailored to each child’s unique anatomical needs, leading to improved outcomes in both function and aesthetics. Over the past 100 years, the surgical literature has placed considerable focus on the comprehensive management of cleft lip [3,9,10]. However, a detailed review of the most cited cleft repair studies is clearly lacking. 

The citation count of a published article serves as an indicator of its significance within a specific field of practice. Articles with higher citation numbers not only enhance the reputation of their authors but also play a critical role in a journal’s standing. The more often articles from a journal are cited, the greater the journal’s impact factor (IF), which reflects its influence and prominence in the academic community [11]. 

Bibliometrics provides a powerful tool for assessing the influence of scholarly work by analyzing publication trends and citation patterns. By examining the content and citations of journal articles, bibliometric analysis helps identify important research topics, influential studies, and key contributors to a field. Researchers have used this approach to evaluate the historical impact of studies, compile recommended reading lists, and uncover gaps in the literature. These insights not only highlight areas of strength but also point to opportunities for further research and improvement [12,13]. One prior bibliometric study explored the field of cleft lip repair, providing a foundation for understanding its scholarly impact [14]. 

However, recent advancements in surgical techniques and a deeper understanding of the condition highlighted the need for an updated analysis. This study aimed to conduct a bibliometric review of the 50 most highly cited publications in cheiloplasty over the past 100 years. Focusing specifically on cleft lip repair, it seeks to identify key trends, influential studies, and notable contributors to the field. Ultimately, this study aimed to provide a comprehensive overview of the most impactful research and offer valuable insights to guide future advancements in cleft care.

## Review

Methodology

Search Strategy 

A comprehensive search was performed using the Web of Science database to identify the most frequently cited publications on cleft lip repair, without any restriction on country of origin, thereby including studies conducted worldwide. The search strategy incorporated the keywords “cleft lip repair” and “cheiloplasty” and was restricted to original research articles and review articles. The results were then filtered to retrieve the top 50 most cited articles, ranked according to their total citation count. These articles were subsequently subjected to bibliometric analysis to evaluate their scientific impact and research trends within the field. The use of a single database ensured consistency and reproducibility in citation-based ranking. However, it is acknowledged that citation counts and article rankings may vary across other databases such as Scopus or Google Scholar, where indexing and citation-tracking methodologies differ.

Study Selection 

Studies were considered for the review if they met the following criteria: They were published without time frame limitation, written in English, focused on cleft lip repair or cheiloplasty, involved human subjects or patient groups with cleft lip, appeared in peer-reviewed journals, and had been cited by other publications. Studies were excluded if they were published in a language other than English, did not primarily focus on cleft lip or cheiloplasty, were published in non-peer-reviewed sources such as conference proceedings, abstracts, posters, editorials, or letters, or had not been cited in other publications.

Data Extraction and Management 

A uniform extraction form in an Excel file was used to extract data from the listed studies. The data collection was overseen by the first author. The filled-out data extraction forms were stored on a password-protected laptop and a USB memory stick for privacy. For every included paper, the following details were noted: article title, authors, journal, year of publication, total number of citations, average number of citations annually, research setting, financial status, study design, evidence strength, primary topic, and use of patient-reported outcome measures (PROMs) for clinical, cosmetic, and other purposes.

Treatment of Missing Data 

In cases where essential information was unavailable within an article, the corresponding authors were contacted to obtain the missing data. When retrieval of such information was not possible, the specific nature of the missing data and its potential implications for the study’s findings were clearly documented and addressed, and these studies were still included in the analysis.

Statistical Analysis

The data was compiled using descriptive statistics. When applicable, the mean ± standard deviation (SD) or median (interquartile range (IQR)) was used to display continuous variables. Frequencies and percentages were used to display categorical variables. IBM SPSS Statistics for Windows, version 25.0 (released 2017, IBM Corp., Armonk, NY) was used for all statistical analyses.

Results

**Figure 3:**
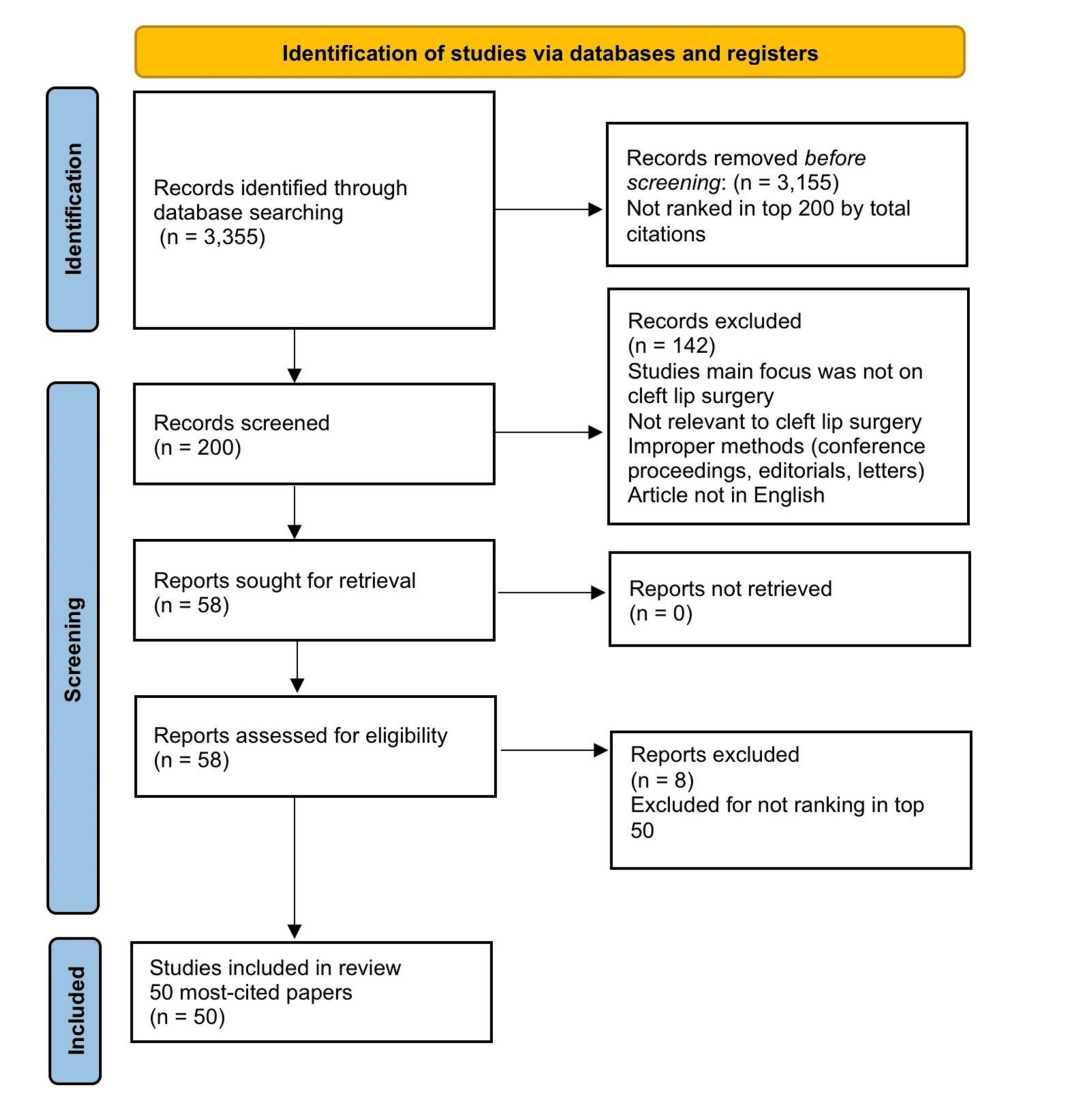
Identification of studies via databases and registers Figure created by authors using Microsoft Word (PRISMA 2020 template)

The selection of the 50 most-cited articles on cleft lip repair followed a structured methodology, commencing with a comprehensive search of the Web of Science database, which initially identified 3,355 publications. To refine this initial pool, the identified articles were then organized and ranked based on their total citation counts. The top 200 articles were selected for detailed screening, while 3,155 records that were not ranked within the top 200 by total citations were removed before further review. During the screening process, 142 publications were excluded. The reasons for exclusion at this stage encompassed several criteria, including if a study’s primary focus was outside the scope of cleft lip surgery, if it lacked relevance to cleft lip surgical procedures, if it employed unsuitable research methodologies (such as abstracts, conference proceedings, editorials, or letters), or if the article was published in a language other than English. A total of 58 reports were then sought for retrieval, none of which were excluded due to retrieval issues (n = 0). Following the eligibility assessment, eight additional articles were excluded for not ranking within the top 50 most-cited publications. Through this rigorous selection process, the 50 most-cited articles were identified and included in the final analysis [6,13-61]. For these 50 articles, a systematic data extraction was performed, capturing key information such as the paper's title, authors, publication year, source journal, total citation count, average citations per year, study setting, funding sources, study design, level of evidence, main subject area, and the utilization of clinical, cosmetic, and PROMs (Figure 3).

**Figure 4:**
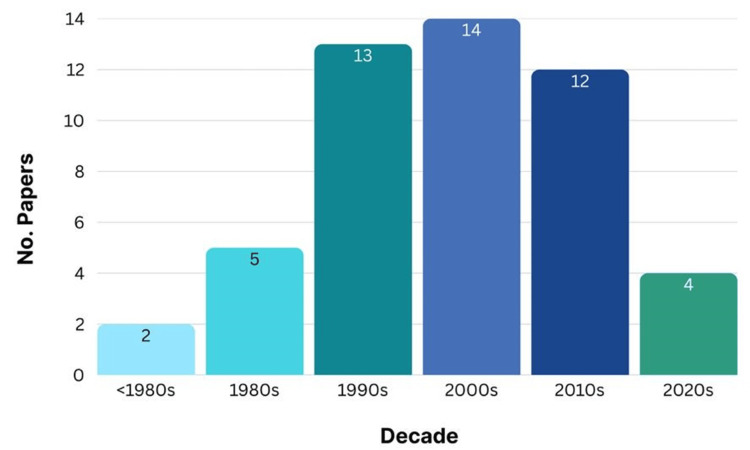
Count of articles published in each decade among the 50 most cited articles Figure created by authors using SPSS version 25

There is a discernible pattern in the research output when the top 50 most cited cleft lip publications from the previous several decades were examined. The decades 1990s and 2000s seemed to have had a particularly significant influence on cleft lip research, as they produced the majority of highly cited articles. This implies that these years probably saw important breakthroughs, well-regarded research, or changes in the focus of the field. Even while the 2010s nonetheless produced a significant number of influential publications, the previous years before 1980 and the more recent data from the 2020s (which only makes up a small portion of the decade) were relatively underrepresented in the top 50 most referenced articles (Figure 4).

**Figure 5:**
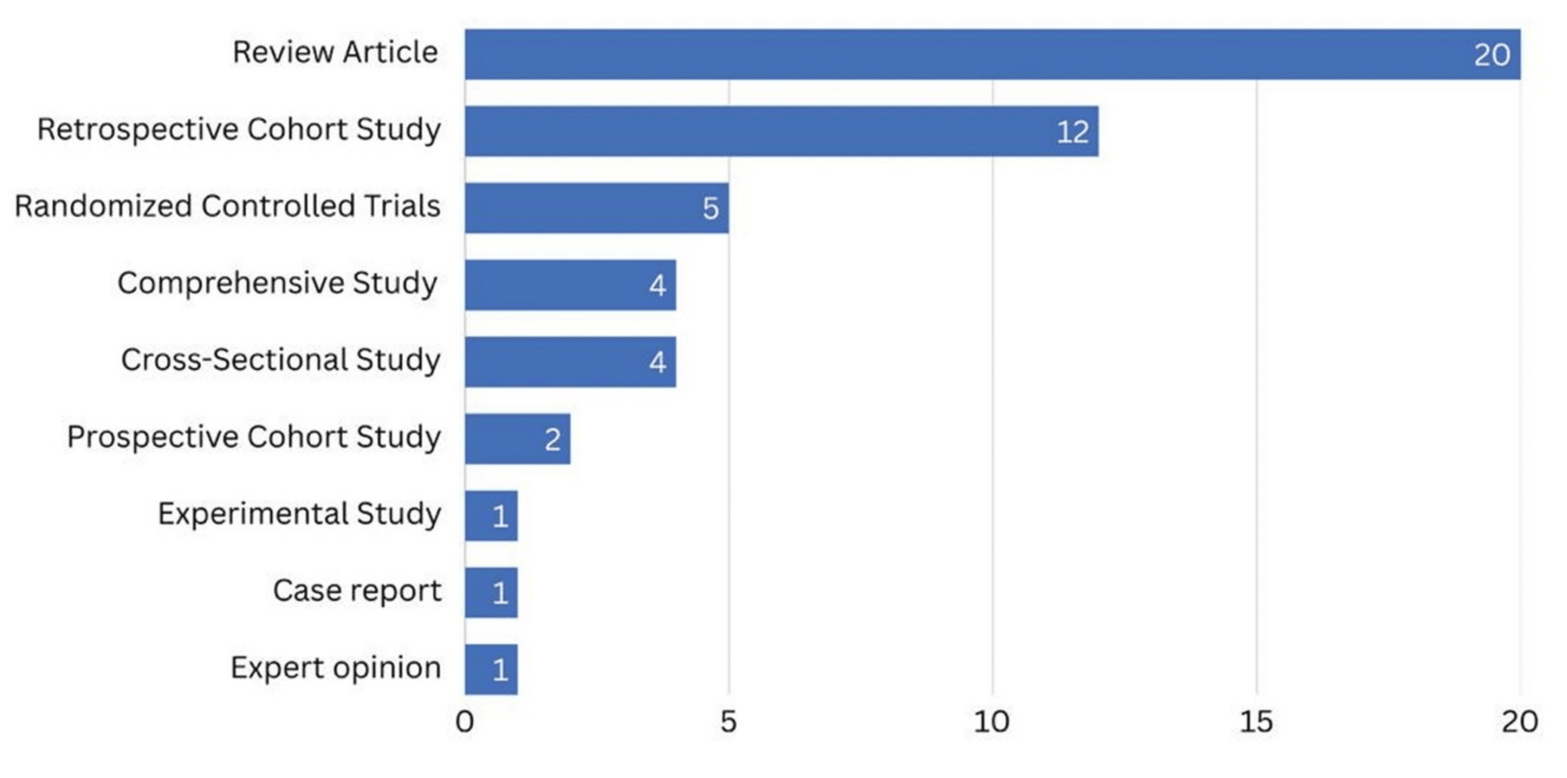
Study designs of the 50 most cited articles on cleft lip repair Figure created by authors using SPSS version 25

The distribution of study designs among the 50 most-cited articles was characterized by a clear dominance of review articles, which make up the largest group with 20 publications. This suggests that the synthesis and summarization of current research play a key role in cleft lip studies. Retrospective cohort studies were the second most common (12 articles), highlighting a considerable dependence on analyzing past information to explore outcomes and patterns in cleft lip treatment. Although randomized controlled trials (RCTs), which are typically considered the benchmark for assessing interventions, were included (five articles), but were less prevalent than observational study designs. Both comprehensive and cross-sectional studies comprised four articles each, while prospective cohort studies were less frequent, comprising only two articles. Experimental studies, case reports, and expert opinions each contributed only one article to the top 50. Overall, the data indicated a primary focus on summarizing current knowledge and analyzing observational data, with fewer original experimental studies (Figure 5).

**Figure 6:**
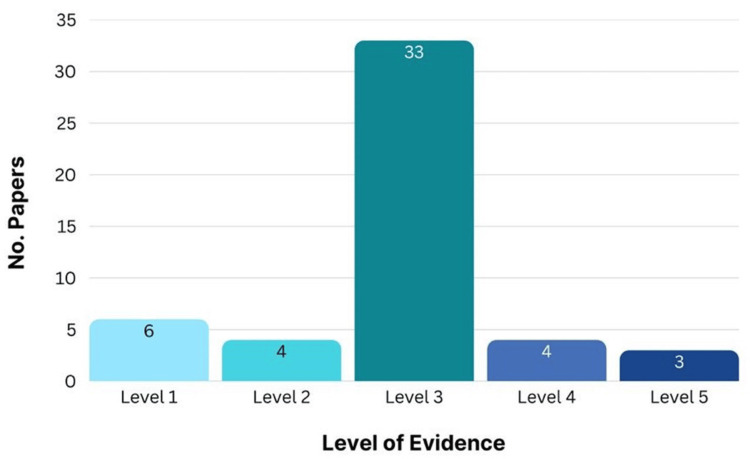
Level of evidence of the 50 most cited articles Figure created by authors using SPSS version 25

According to this distribution, a sizable amount of the important research on cleft lip repair was based on data from carefully planned non-experimental studies, like case-control and observational studies (Level 3). Evidence from individual RCTs (Level 2) and systematic reviews of RCTs (Level 1) was available, but it makes up a lower percentage of the most often cited literature. This collection of significant publications also underrepresented data from systematic reviews of descriptive and qualitative research (Level 5) and well-designed cohort and case-control studies (Level 4) (Figure 6).

**Figure 7:**
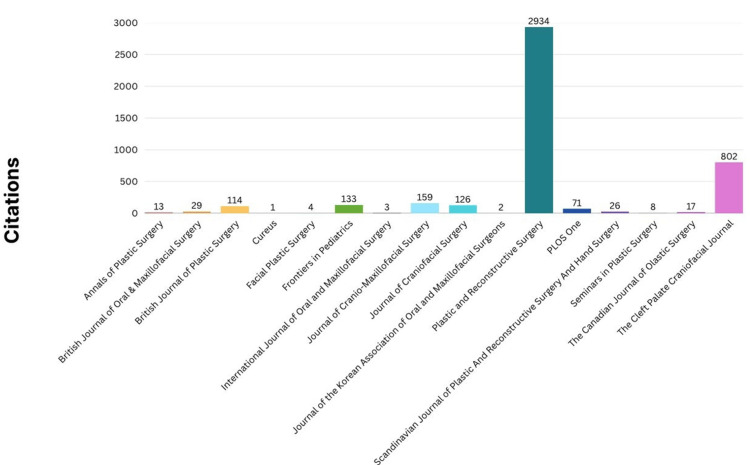
Total citation per journal Figure created by authors using SPSS version 25

A highly skewed distribution of total citations across journals was displayed, with a pronounced dominance of *Plastic and Reconstructive Surgery*. The journal's impressive citation count of 2,934 suggested that it had been a central platform for influential research that has significantly shaped the understanding and practice of cleft lip repair. This high level of citation might indicate that articles published in this journal were frequently referenced by other researchers, potentially due to their methodological rigor, clinical relevance, or the journal's broad readership within the plastic surgery community. By contrast, the majority of journals listed demonstrated a much lower impact, with citation counts below 150. This disparity raises questions about the factors that contribute to the varying levels of influence among journals in this specialized field. The *Cleft Palate-Craniofacial Journal*, with 802 citations, represented a notable secondary hub of influential publications, suggesting its importance as a specialized outlet for cleft-related research. However, the overall trend indicated that a substantial portion of the most highly cited cleft lip research was concentrated within a select few journals, particularly *Plastic and Reconstructive Surgery* (Figure 7).

With 15 articles, surgical technique was the most frequently discussed subject, indicating a strong emphasis on operative methods within this research. Nasal deformity correction was also a key subject, with six articles. Several subjects had a moderate number of articles: four each for bilateral cleft lip repair, complications and revisions, and adjunctive treatments and three each for muscle repair and outcomes, and assessment. Anatomy was covered in two articles, comprehensive care in three articles, and psychology in one article (Table 1).

**Table 1:** Main subjects of the 50 most-cited articles

Main subject	Number of papers
Surgical technique	15
Nasal deformity correction	6
Bilateral cleft lip repair	4
Muscle repair	3
Complications and revisions	4
Outcomes and assessment	3
Adjunctive treatments	4
Anatomy	2
Comprehensive care	3
Psychology	1

Several significant patterns and insights into the development of cleft lip restoration methods and thoughts were revealed by the examination of these extremely influential papers. Notably, many adaptations and improvements of well-known techniques, like the Millard rotation-advancement, were regularly examined and evaluated for their ability to produce better functional and aesthetic results. Numerous studies highlighted the advantages of primary nasal reconstruction and the application of methods such as nasoalveolar molding, underscoring the need to treat both lip and nasal abnormalities. The move toward a multidisciplinary and holistic approach to cleft care was also highlighted, recognizing the need for long-term monitoring and the effect of treatment on patients' quality of life. The inclusion of research on subjects like the use of adjuvant therapies (like botulinum toxin) and postoperative problems (like fistula incidence) showed the continuous attempts to improve patient outcomes and reduce side effects. A quantifiable indicator of the impact and influence of this research in the field of cleft lip repair was provided by bibliometric statistics, such as total citation counts. One can better grasp the significant developments and ongoing difficulties in the search for the best cleft lip. The treatment was informed by examining the main findings of these widely cited publications (Table 2).

**Table 2:** Key Information and primary outcomes of the top-cited cleft lip repair studies sorted by the total number of citations

Title	Year of publication	Sample size (number of cases)	Total number of citations	Primary outcome
Cleft Lip: A Comprehensive Review [8]	2013	N/A	133	A multidisciplinary team approach is crucial for optimal orofacial cleft management, requiring long-term follow-up to assess functional, aesthetic, and quality-of-life outcomes.
Unilateral Cleft Lip Repair: An Anatomical Subunit Approximation Technique [15]	2005	N/A	329	This surgical technique for unilateral cleft lip repair focuses on aligning lip elements along natural borders to improve aesthetics by reducing scars and maintaining natural contours.
Long-Term Effects of Nasoalveolar Molding on Three-Dimensional Nasal Shape in Unilateral Clefts [16]	1999	10	328	Presurgical nasoalveolar molding effectively treats cleft lip nasal deformity and significantly improves nasal symmetry.
Presurgical Columellar Elongation and Primary Retrograde Nasal Reconstruction in One-Stage Bilateral Cleft Lip and Nose Repair [17]	1998	N/A	292	A combination of surgical and non-surgical approaches effectively treats bilateral cleft lip and palate and congenital ear deformities, respectively.
Primary Repair of the Unilateral Cleft Lip Nose: Completion of a Longitudinal Study [18]	1996	10	269	McComb primary cleft nose correction effectively resolves nasal deformity in unilateral cleft lip cases, ensuring long-term stability and supporting normal nasal growth.
Reverse-U Incision for Secondary Repair of Cleft Lip Nose [19]	1977	N/A	264	The reverse-U incision is an effective technique for secondary cleft lip nose repair, especially for underdeveloped cartilage and thick skin.
The Principle of Rotation Advancement for Repair of Unilateral Complete Cleft Lip and Nasal Deformity: Technical Variations and Analysis of Results [20]	1999	105	227	The refined Millard rotation-advancement technique enhances nasolabial symmetry and lowers revision rates, contingent on surgical skill and comprehensive care.
The Repair of the Unilateral Cleft Lip by the Stencil Method [21]	1946	N/A	206	A straightforward surgical technique effectively repairs unilateral cleft lip deformities, improving muscle alignment, skin coverage, and lip anatomy.
Fistula Incidence After Primary Cleft Palate Repair: A Systematic Review of the Literature [22]	2014	9294	132	Postoperative oronasal fistulae are a significant concern in cleft palate repair, with the type of cleft and surgical technique influencing their occurrence.
Unilateral Cleft Lip Repair [23]	1987	N/A	126	The modified Millard rotation advancement procedure is an effective technique for unilateral cleft lip repair. The procedure results in a more symmetrical scar and improved lip length.
Repair of Cleft Lip with Nonsurgical Correction of Nasal Deformity in the Early Neonatal Period [24]	1989	44	118	Nonsurgical correction of cleft lip nasal deformity in newborns yields better short-term results than surgery at 3 months due to the nose's malleability.
The Orbicularis Oris Muscle: A Functional Approach to Its Repair in the Cleft Lip [25]	1983	N/A	114	Muscle reconstruction is vital in cleft lip repair, and the rotation advancement technique effectively achieves proper anatomy and function.
Principles and Techniques of Bilateral Complete Cleft Lip Repair [26]	1985	N/A	113	Two-stage cleft lip repair yields natural-looking lip and nose shapes, and while prolabium widening may occur, complete primary muscle repair can minimize it.
Repair of Bilateral Cleft Lip: Review, Revisions, and Reflections [27]	2003	50	110	Successful bilateral cleft lip and palate (BCLP) treatment relies on correct premaxillary positioning, single-stage closure, and consistent monitoring, with potential adolescent maxillary advancement.
Current Surgical Practices in Cleft Care: Unilateral Cleft Lip Repair [28]	2008	N/A	109	Surgical and non-surgical techniques, often involving multidisciplinary care and long-term follow-up, aim to improve aesthetics, function, and quality of life in various cleft lip and palate deformities, with a focus on minimizing scarring, maintaining symmetry, and supporting normal growth.
In Utero Cleft Lip Repair in A/J Mice [29]	1985	N/A	107	In utero microsurgery in mice shows promise for cleft lip repair, resulting in near-normal appearance and scar-free healing at birth.
Unilateral Cleft Lip-Nose Repair: A 33-Year Experience [30]	2003	N/A	106	Primary lip and nasal reconstruction in the initial surgery is the standard for unilateral cleft lip and palate, enhancing symmetry and yielding consistent aesthetic and functional results.
Primary Repair of Bilateral Cleft Lip and Nasal Deformity [31]	2001	N/A	103	Careful manipulation of nasal cartilage aims for symmetry in cleft lip repair, but risks like tissue loss and hypertrophic scarring may necessitate additional surgery.
Primary Repair of the Bilateral Cleft Lip Nose: A 15-Year Review and a New Treatment Plan [32]	1990	N/A	102	A two-stage cleft lip repair, initially facing issues like a long columella and broad nasal tips, was improved by reconstructing the columella with nasal tip tissue.
Correction of the Bilateral Cleft Lip Nasal Deformity: Evolution of a Surgical Concept [33]	1992	N/A	95	The evolution of bilateral cleft lip nasal repair favors simultaneous lip and nose correction, emphasizing precise cartilage positioning and premaxilla alignment to optimize aesthetics, though postoperative distortion remains a potential complication.
Bilateral Complete Cleft Lip and Nasal Deformity: An Anthropometric Analysis of Staged to Synchronous Repair [34]	1995	N/A	93	Synchronous repair of bilateral complete cleft lip and nasal deformity yields better anatomical outcomes compared to staged repair, though meticulous alignment is crucial to avoid nasal distortion.
Lip Height and Lip Width After Extended Mohler Unilateral Cleft Lip Repair [35]	2003	120	93	Extended Mohler unilateral cleft lip repair achieves favorable aesthetic outcomes, improving lip width symmetry without causing a short lip.
Nonsurgical Correction of Nasal Deformity in Unilateral Complete Cleft Lip: A 6-Year Follow-Up [36]	1999	91	87	Early nonsurgical nasal correction with a prosthesis in unilateral cleft lip patients provides lasting improvements in nasal symmetry and columellar length, often reducing the need for primary surgery.
Quantitative 3D Soft Tissue Analysis of Symmetry Prior to and After Unilateral Cleft Lip Repair Compared With Non-Cleft Persons [37]	2008	11	79	Quantitative 3D analysis confirms significant facial symmetry improvements after unilateral cleft lip repair, establishing it as an effective outcome measurement tool.
The Use of Nasal Splints in the Primary Management of Unilateral Cleft Nasal Deformity [38]	1999	30	78	Postoperative nasal splinting after primary unilateral cleft lip and nose repair helps maintain the corrected nasal shape, leading to a significantly better aesthetic outcome.
Importance of Muscle Reconstruction in Primary and Secondary Cleft Lip Repair [39]	1979	N/A	75	Proper muscle reconstruction is crucial in both primary and secondary cleft lip repair for improved aesthetics and function; inadequate repair can lead to functional and cosmetic issues.
Botulinum Toxin to Improve Results in Cleft Lip Repair [40]	2014	59	74	Botulinum toxin shows promise as a supplementary treatment in cleft lip repair by improving lip symmetry and reducing postoperative scarring.
Photogrammetric Comparison of Two Methods for Synchronous Repair of Bilateral Cleft Lip and Nasal Deformity [41]	1998	25	71	Mulliken's method in synchronous cleft lip and nose repair yielded better nasal tip projection and columellar length compared to Trott's method, both showing significant improvements but with potential for asymmetry and abnormal nasal dimensions.
Botulinum Toxin to Improve Results in Cleft Lip Repair: A Double-Blinded, Randomized, Vehicle-Controlled Clinical Trial [42]	2014	58	71	A double-blind study confirms that botulinum toxin injections significantly improve cosmetic outcomes in cleft lip repair by reducing scarring and enhancing lip symmetry.
The Anatomy of Cupid’s Bow in Normal and Cleft Lip [43]	1993	N/A	70	Precise anatomical alignment of the Cupid's bow during cleft lip surgery is crucial for restoring natural lip contours, as detailed anatomical analysis reveals key differences between normal and cleft lips.
Fifty Years of the Millard Rotation-Advancement: Looking Back and Moving Forward [44]	2009	N/A	65	The Millard rotation-advancement technique aims to restore lip anatomy in cleft repair through precise incisions and tissue manipulation, but potential complications include scarring, asymmetry, and challenges in nasal correction.
Analysis of the Cleft Lip Nose in Submental-Vertical View, Part I: Reliability of a New Measurement Instrument [45]	2007	N/A	65	A new nasal analysis method, assessing internal features and facial positioning, proves effective for comparing surgical outcomes using indirect anthropometry.
CAD/CAM Silicone Simulator for Teaching Cheiloplasty: Description of the Technique [46]	2014	N/A	29	Silicone simulators offer a realistic and affordable training method for cheiloplasty, allowing trainees to practice various cleft lip repairs with a readily manufacturable tool.
Bilateral Infraorbital Block With 0.5% Bupivacaine in Children as Post-Operative Analgesia Following Cheiloplasty [47]	1991	60	26	Bilateral infraorbital nerve block with bupivacaine effectively provides prolonged pain relief in children post-cleft lip repair, easing nursing care and improving parental well-being.
Unilateral Cleft Lip Repair [48]	2014	N/A	25	A comprehensive approach, especially with the rotation-advancement technique by expert surgeons using standardized measurements and quality assurance, is essential for achieving optimal long-term results in cleft lip and palate treatment.
Use of Botulinum Toxin in Cheiloplasty: A New Method to Decrease Tension [49]	2009	5	17	Intraoperative botulinum toxin injection safely reduces orbicularis oris muscle activity in children with cleft lip and palate.
Follow-Up of Unilateral Cleft Lip Nose Deformity After Secondary Repair With a Modified Reverse-U Method [50]	2011	89	16	The modified reverse-U method effectively achieves long-term nasal dome symmetry in secondary unilateral cleft-lip nasal repair, demonstrating Excellent or Good results in over 81% of cases, even after multiple prior surgeries.
Correction of Secondary Vermilion Notching Deformity in Unilateral Cleft Lip Patients: Complete Revision of Two Errors [51]	2011	104	11	Lengthening the central lip's oral lining and adjusting the cleft-side Cupid's bow height to the thickest vermilion effectively corrects secondary notching deformity.
Important Aspects of Oral Lining in Unilateral Cleft Lip Repair [52]	2009	389	9	Considering the oral lining in cleft lip repair allows for an aesthetically pleasing lip with central fullness and slight eversion, according to the authors' methods.
Revisional Techniques for Secondary Cleft Lip Deformities [53]	2021	N/A	8	Secondary deformities after cleft lip repair are common and require a spectrum of treatments, emphasizing accurate assessment and appropriate surgical techniques for optimal cosmetic results with minimal intervention.
Modified Design of Cupid’s Bow in the Repair of Unilateral Microform Cleft Lip: In Case of Deficient Distance Between the Midline and the Cleft Side Cupid’s Bow Peak [54]	2009	12	7	A modified Cupid's bow design effectively creates a symmetric Cupid's bow and natural philtrum in unilateral microform cleft lip patients, though long-term results require further observation.
Practical Repair Method for Unilateral Cleft Lips: Straight-Line Advanced Release Technique [55]	2016	145	5	The StART technique is a novel, intuitive, and effective method for unilateral cleft lip repair, yielding a natural, balanced lip with a straight scar and a low need for secondary operations, even for less experienced surgeons.
Anatomical Reconstruction of the Nasal Floor in Complete Unilateral Cleft Lip Repair [56]	2017	72	5	Nasal floor reconstruction is vital for successful complete unilateral cleft lip repair, and the StART technique reliably creates a natural, symmetrical floor with minimal scarring.
Open Versus Closed Rhinoplasty With Primary Cheiloplasty: A Comparative Study [57]	2012	36	4	Open rhinoplasty significantly impacts alar base width and might be more effective for nasal correction in unilateral cleft lip, though no definitive advantage over closed rhinoplasty was established in this study, possibly due to sample size and age.
Reconstruction of Bilateral Cleft Lip and Nose Deformity [58]	2008	N/A	4	Specific surgical techniques involving mucosal and muscle flaps, along with cartilage repositioning, effectively reconstruct bilateral cleft lip and nose deformities for improved appearance and function.
Unilateral Cleft Lip Repair by Rotation/Advancement: Potential Errors and How to Avoid Them [59]	2007	N/A	4	Successful cleft lip repair hinges on meticulous surgical technique and attention to detail, with surgeons needing to be keenly aware of potential pitfalls during flap creation.
Revisiting Straight-Line Repair in Unilateral Complete Cleft Lip: A Comparison With Rotation-Advancement Repair [60]	2021	71	3	The SLR-ml technique offers an effective and reliable approach to unilateral cleft lip repair, yielding symmetrical results with minimal scarring and avoiding short lip, comparable to the RAR technique.
Three-Dimensional Measurement of the Lateral Lip Element Sacrificed in Primary Repair of a Unilateral Cleft Lip [61]	2020	50	3	Primary lip and nasal reconstruction during the first surgery is the standard for unilateral cleft lip and palate, improving symmetry and achieving consistent aesthetic and functional results.
Importance of Various Skin Sutures in Cheiloplasty of Cleft Lip [62]	2018	N/A	2	Inner muscular reorientation is the most important in the cheiloplasty of cleft lip patients for minimizing postoperative complications and the esthetic outcomes, regardless of skin suture materials.
Analysis of Different Facets of the Rule of 10 for Cleft Lip Repair for Their Application in the Current Era [63]	2024	N/A	1	The rule of 10 is not considered a gold standard by most of the centers in India, and the decision-making was based on the overall physiological status of the patients, the experience of the surgeon, and the anesthetic and post-operative care facilities available at the center.

Discussion

A cleft lip is a common congenital anomaly caused by incomplete fusion of the facial processes in the upper lip during early gestation, typically between the fourth and seventh week, affecting about one in 700 births. This condition results from a combination of genetic factors, including an increased inheritance risk in affected families, and environmental influences [6,64,65].

A bibliometric analysis of the 50 most-cited cleft lip publications highlighted how the field has evolved over 100 years, driven by innovations in surgical techniques, anesthesia advancements, and integrated care models that together have enhanced safety, functional outcomes, and aesthetics [66,67]. This analysis reflected a past dependence on merging observational data and existing knowledge instead of relying on prospective, high-evidence trials [68]. This is further supported by the distribution of levels of evidence, as most highly cited articles were based on Level 3 evidence (case-control and observational studies), with fewer contributions from Level 1 (RCTs and systematic reviews of RCTs) [69].

A huge gap in evidence-based surgical decision-making was highlighted by the diminished number of RCTs, despite the fundamental role of such studies. This pattern underscores the need for more high-quality, prospective research in cleft lip repair to strengthen the evidence base and inform best practices [68].

Interestingly, the 1990s and 2000s saw a surge in significant publications, which coincided with significant developments in surgical methods like nasoalveolar molding (pre-surgical technique) and the Millard rotation-advancement. These methods, which are commonly utilized, showed how the field emphasizes both cosmetic and functional results. Moreover, the practical outcomes and the aesthetic refinement are both emphasized by these strategies, which are highly prevalent in the sector [28]. In addition to the Millard rotation-advancement technique, other widely used and well-known surgical methods for unilateral cleft lip repair include the Modified Mohler, the Tennison-Randall triangular flap technique, and Fisher’s straight-line repair. Each offers distinct advantages and is used based on specific clinical scenarios.

The Millard rotation-advancement technique, introduced in the 1950s, is the most prevalent worldwide and is valued for its versatility, adaptability, and the way it preserves the natural anatomy of the lip and philtrum. It allows surgeons to make intraoperative adjustments, remove minimal tissue, and achieve excellent symmetry, making it suitable for a wide range of cleft severities [58,70,71]. The modified Mohler technique is a direct modification of Millard’s method, extending the incision into the columella and using a C-flap to achieve greater vertical lip length and improved philtral symmetry, especially in wide or severe clefts. The scar in Mohler’s method follows natural subunit borders, making it less conspicuous and more aesthetically pleasing [23,58,72-74].

The Tennison-Randall triangular flap technique is particularly favored for wide clefts with significant vertical deficiency; it uses a geometric triangular flap to lengthen the medial lip, resulting in a zigzag scar that is well camouflaged and less prone to hypertrophy. This method is highly reproducible and reliable, especially where precise vertical lengthening is needed [75].

The Fisher anatomical subunit approximation technique, developed in 2005, aligns scars with the natural subunits of the lip, improving aesthetic outcomes and reducing scar derangements and revision rates. Studies have shown that the Fisher technique can produce more desirable lip aesthetics and better nasolabial symmetry than either Millard or Mohler in some cases.

Straight-line repairs (such as Rose-Thompson and Spina’s techniques) are now less commonly used due to their association with lip shortening, blunted Cupid’s bow, and higher risk of scar contracture, but they might still be chosen for select cases or certain regions [72,76]. Importantly, the modified Mohler technique is a refinement of the original Millard method. Millard developed the foundational rotation-advancement approach, and Mohler later improved it by addressing some of its limitations, particularly in achieving better vertical lip length and more natural scar placement [23,58,74]. Despite the availability of newer or more specialized techniques like Mohler or Fisher, the Millard technique remains the most prevalent because of its proven reliability, flexibility, and consistently excellent functional and cosmetic results. Surgeons often favor Millard’s approach due to its adaptability to a wide variety of cleft presentations and its long-standing track record of success, even as modifications and alternative methods have emerged to address specific challenges or aesthetic preferences [58,70,71].

One of the main limitations of this research was the restricted accessibility of several relevant studies, as many were classified as closed access. This might have limited the comprehensiveness of the data collection and excluded potentially valuable insights. Additionally, despite efforts to ensure objectivity, it was acknowledged that bias, both in the original studies and in the selection process, could not be eliminated. Furthermore, this analysis was based solely on the Web of Science database, which was chosen for its standardized citation metrics and comprehensive coverage of peer-reviewed journals. While this ensured consistency and reproducibility in ranking the most-cited publications, citation counts and article indexing may vary across other databases such as Scopus or Google Scholar, potentially leading to minor variations in the relative ranking of highly cited papers. These limitations might affect the generalizability and balance of current review findings and should be considered when interpreting the results.

Future research should prioritize randomized trials and prospective cohort studies, including PROMs. This transition is crucial for augmenting evidence quality, bolstering clinical recommendations, and eventually enhancing patient outcomes. This study identified trends and gaps in the literature, serving as a resource for academics and doctors aiming to enhance standards in cleft lip restoration. To address the limitations identified in this study, future research should aim to overcome the barrier of restricted access by incorporating a broader range of open-access databases and collaborative networks that ensure comprehensive data inclusion.

In addition, efforts must be made to minimize bias by employing standardized selection criteria methodologies. Given the evident gap in high-level evidence, particularly the scarcity of RCTs and prospective cohort studies, future investigations should prioritize these designs to improve the robustness and generalizability of findings. Moreover, research objectives should expand beyond surgical techniques to encompass holistic, patient-centered outcomes, especially those reflecting psychological well-being and PROMs, which are currently underrepresented in the literature. By addressing these limitations and gaps, future studies can enhance the quality of evidence, guide clinical decision-making, and ultimately contribute to more effective and empathetic cleft lip care.

## Conclusions

This study performed a bibliometric analysis of the 50 most influential publications in cleft lip surgery over the previous 100 years, identifying the most prominent research trends, journals, and contributors to the field. The findings revealed that influential research was concentrated in the 1990s and 2000s, with a focus on review articles and retrospective studies and a notable paucity of high-level evidence. These results underscore the need for more rigorous research in the future, with an emphasis on clinical trials and prospective studies, and the inclusion of patient outcome indicators to raise the standard of surgical care.
